# *Drosophila* KCNQ Channel Displays Evolutionarily Conserved Electrophysiology and Pharmacology with Mammalian KCNQ Channels

**DOI:** 10.1371/journal.pone.0023898

**Published:** 2011-09-07

**Authors:** Sonia Cavaliere, James J. L. Hodge

**Affiliations:** University of Bristol, School of Physiology and Pharmacology, Medical Sciences Building, Bristol, United Kingdom; Yale School of Medicine, United States of America

## Abstract

Of the five human KCNQ (Kv7) channels, KCNQ1 with auxiliary subunit KCNE1 mediates the native cardiac I_Ks_ current with mutations causing short and long QT cardiac arrhythmias. KCNQ4 mutations cause deafness. KCNQ2/3 channels form the native M-current controlling excitability of most neurons, with mutations causing benign neonatal febrile convulsions. *Drosophila* contains a single KCNQ (dKCNQ) that appears to serve alone the functions of all the duplicated mammalian neuronal and cardiac KCNQ channels sharing roughly 50–60% amino acid identity therefore offering a route to investigate these channels. Current information about the functional properties of dKCNQ is lacking therefore we have investigated these properties here. Using whole cell patch clamp electrophysiology we compare the biophysical and pharmacological properties of dKCNQ with the mammalian neuronal and cardiac KCNQ channels expressed in HEK cells. We show that *Drosophila* KCNQ (dKCNQ) is a slowly activating and slowly-deactivating K^+^ current open at sub-threshold potentials that has similar properties to neuronal KCNQ2/3 with some features of the cardiac KCNQ1/KCNE1 accompanied by conserved sensitivity to a number of clinically relevant KCNQ blockers (chromanol 293B, XE991, linopirdine) and opener (zinc pyrithione). We also investigate the molecular basis of the differential selectivity of KCNQ channels to the opener retigabine and show a single amino acid substitution (M217W) can confer sensitivity to dKCNQ. We show dKCNQ has similar electrophysiological and pharmacological properties as the mammalian KCNQ channels, allowing future study of physiological and pathological roles of KCNQ in *Drosophila* and whole organism screening for new modulators of KCNQ channelopathies.

## Introduction

Voltage-gated potassium (Kv) channels form a diverse gene family with 40 members in humans divided into 12 subfamilies. Because mutations in over sixty channel genes are already known to result in human disease, developing viable genetic models to study individual ion channel functions and channelopathies is of increasing clinical importance [1], [2]. KCNQ channels are a particular hotspot of genetic diseases reflecting the range of important physiological roles they mediate. KCNQ1 assembles with KCNE1 auxiliary subunits in cardiac muscle to produce the native slowed delayed rectifier current (I_KS_). Mutations of KCNQ1 cause a number of life threatening diseases, for instance, loss of function mutations cause the common cardiac arrhythmia Long QT syndrome associated with increased risk of Torsades des Pointes arrhythmia and sudden death. Some KCNQ1 loss of function mutations, result in Jervell and Lange-Nielsen syndrome which involve cardiac and auditory defects. Conversely, KCNQ1 gain of function mutations result in hastening of the cardiac action potential repolarization causing Short QT syndrome and atrial fibrillation. KCNQ1 is a major target of anti-arrhythmic drugs, with blockers prolonging repolarization hence treating Short QT syndrome and openers speeding repolarization in Long QT syndrome [1]–[4]. Recently KCNQ1 mutations have also been found to be strongly associated with Type II (adult onset) diabetes [5], [6].

KCNQ2-5 are expressed in the nervous system with KCNQ2 and KCNQ3 heteromultimerising to form the main channel mediating the M-current (named after its suppression by muscarinic acetylcholine receptor agonists), a slowly activating and slowly deactivating potassium (K^+^) current open in the voltage range required for action potential generation therefore determining neuronal excitability in most neurons. Loss of functions mutations in either KCNQ2 or KCNQ3 result in a form of epilepsy called benign neonatal febrile convulsions [7]. KCNQ5 is highly expressed in hippocampus and cortex and may contribute to M-current and excitability in these brain regions by heteromultimerising with KCNQ3 [8]. KCNQ4 is expressed in the cochlea and is important for auditory physiology with loss of function mutations result in autosomal dominant deafness and age-related hearing impairment [9], [10]. M-current inhibitors such as linopirdine and XE-991 increase excitability and have been shown to improve learning and memory in some animal models including those for dementia while neuronal KCNQ channel specific openers such as retigabine are anticonvulsants and flupirtine is used clinically as an analgesic [2], [11], [12]. The involvement of KCNQ1 in type II diabetes emphasises the importance of KCNQ channel expression and function in cells other than cardiac myocytes and neurons [13].

As opposed to the five KCNQ channels in mammals, *Drosophila* has a single KCNQ channel [14] that is expressed broadly in both the nervous system and heart [15], [16] and its genome does not contain any *KCNE* genes [14]. Expression of *Drosophila KCNQ* (*dKCNQ*) in Chinese Hamster Ovary (CHO) cells showed that it encodes a voltage-sensitive K^+^ current that activates and deactivates slowly becoming active in the sub-threshold voltage range (from about −60 mV) similar to the M-current. It was also shown that dKCNQ mediates an M-current because it was suppressed by activation of a muscarinic receptor [15].

dKCNQ has been shown to have a important cardiac function, which was shown to be age-dependent. *dKCNQ* levels were shown to decrease in aging heart in parallel with an increase in incidence of cardiac arrhythmias and dysfunction. Hearts from young *dKCNQ* null flies already have the prolonged contractions and fibrillations similar to Torsades des Pointes arrhythmias and as seen in aged flies [16].

In order to study the function of KCNQ channels further and to help develop a genetic model for KCNQ channelopathies we have characterised the electrophysiological and pharmacological properties of *Drosophila* KCNQ. We perform the first detailed comparison of dKCNQ to mammalian cardiac and neuronal KCNQ channels in terms of their electrophysiological properties and characterise the effect of a range of clinically relevant pharmacological KCNQ blockers and openers.

## Results

### dKCNQ current is similar to the neuronal M-current mediated by KCNQ2/3 channel but has some features of the cardiac KCNQ1/KCNE1 (I_Ks_) current

**Figure 1:**
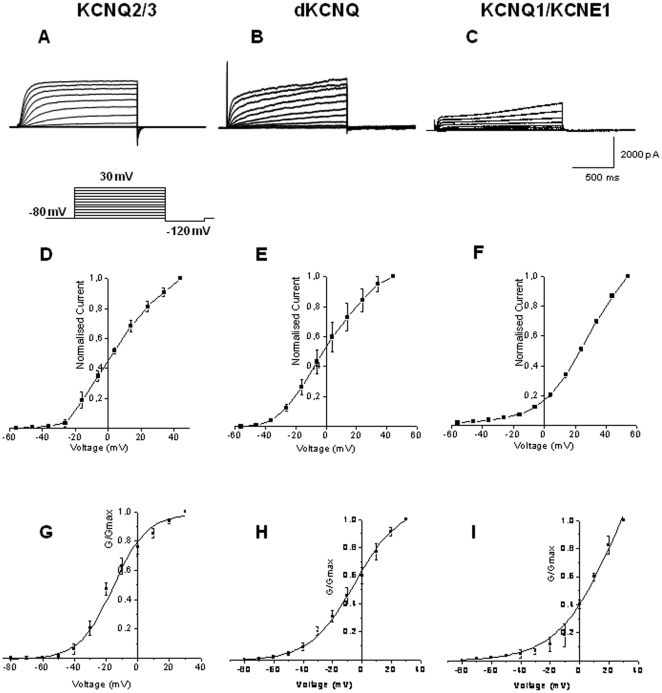
The *Drosophila* KCNQ (dKCNQ) current is most similar to neuronal KCNQ2/3 but shows some features of the cardiac KCNQ1/KCNE1 current. Representative traces of (A) KCNQ2/3, (B) dKCNQ and (C) KCNQ1/KCNE1 currents. HEK cells were held at −80 mV for 100 ms and then depolarized in 10 mV increments from −80 mV to +30 mV for 1.3 sec for KCNQ2/3 and dKCNQ respectively. Cells expressing KCNQ1/KCNE1 were depolarized from −80 mV to +40 mV. After 2.5 sec the cells were stepped down to −120 mV for 125 ms. Current over capacitance at a given voltage is given for (D) KCNQ2/3, (E) dKCNQ and (F) KCNQ1/KCNE1 currents. G-V curves for (G) KCNQ2/3, (H) dKCNQ and (I) KCNQ1/KCNE1 currents. dKCNQ n = 5, KCNQ2/3 n = 5 and KCNQ1/KCNE1 n = 4.

The single *Drosophila* KCNQ channel shows a high level of amino acid identity with the human KCNQ channels: KCNQ5 (amino acid identity, 64%), KCNQ4 (61%), KCNQ2 (57%), KCNQ1 (47%) and KCNQ3 (46%). Previous studies have reported both neuronal and cardiac expression of *dKCNQ* [15], [16], but absence of *KCNE* genes in *Drosophila* [14]. In order to make a detailed electrophysiological and pharmacological comparison between dKCNQ and mammalian neuronal KCNQ2/3 and cardiac KCNQ1/KCNE1 currents, we expressed the respective channels in Human Embryonic Kidney (HEK) cells, comparison of this combination of subunits was chosen as these are the subunits expected in the channel complexes in native *Drosophila* [14]–[16], mammalian neuronal [7], [13], [17], [18] and cardiac [3], [4], [19] tissue respectively. Whole cell voltage clamp recordings revealed dKCNQ (Figure 1B) showed many of the features of the M-current encoded by mammalian KCNQ2/3 channels (Figure 1A), including activation in a range likely to be subthreshold for action potential firing (Figure 1D–E and G–H; Table 1). Conductance-voltage (G-V) curves show that dKCNQ reaches half maximal activation around *V_0.5_* = −4.9±3.4 mV (n = 5) around the value (p>0.05) for KCNQ2/3 (*V_0.5_* = −15.3±1.4 mV, n = 5), with the cardiac KCNQ1/KCNE1 (Figure 1C, F and I; Table 1) current activating at more depolarized voltages (*V_0.5_* = 23.9±7.1 mV, p<0.05, n = 4). The slope factor (Table 1), a measure of the depolarization required for an *e*-fold change in conductance is 15.2±1.9 mV for dKCNQ which is most similar and not significantly different (p>0.05) to KCNQ2/3 (10.6±1.0 mV) but again intermediate in value between the mammalian neuronal M-current and the cardiac KCNQ1/KCNE1 current (19.4±1.2 mV) which also was not significantly different from the dKCNQ slope factor (p>0.05). The activation can be described by a sum of two exponentials (Table 1), with time constants of 78.6±15 and 1665±685 ms for dKCNQ at 0 mV. The deactivation can be also described by sum of two exponentials with the time constant of 30.3±14.0 ms for the slow component and 14.8±4.4 ms for the fast component at 0 mV (n = 5). For KCNQ2/3 the activation can be described by a sum of two exponentials (Table 1), with time constants of 80.3±25 and 254±42 ms at 0 mV and a single deactivation time constant of 17.2±1.2 ms at 0 mV (n = 5). While the KCNQ1/KCNE1 activation can be described by a sum of two exponentials with time constants of 1188±180 and 1752±578 ms at 0 mV and a single exponential of 10.0±1.1 ms at 0 mV for the deactivation curve (n = 4).The dKCNQ fast activation component was significantly different (p<0.05) to the KCNQ1/KCNE1 fast activation component but similar (p>0.05) to the KCNQ2/3 fast activation. Conversely the dKCNQ slow activation component was significantly (p<0.05) different to KCNQ2/3 slow activation component although more similar (p>0.05) to the KCNQ1/KCNE1 slow activation component. There was no significant (p>0.05) difference between the three different deactivation components.

**Table 1:** *Drosophila* KCNQ kinetics are intermediate between those of the mammalian neuronal KCNQ2/3 and cardiac KCNQ1/KCNE1 current, but are more similar to KCNQ2/3.

	KCNQ2/3	dKCNQ	KCNQ1/KCNE1
V_0.5_ (mV)	−15.3±1.4	−4.9±3.4	23.9±7.1
Slope factor	10.6±1.0	15.2±1.9	19.4±1.2
Activation τ (ms)			
Fast:	80.3±25	78.6±15	1188±180
Slow:	254±42	1665±685	1752±578
Deactivation τ (ms)	17.2±1.2	14.8±4.4	10.0±1.1
		30.3±14	
n	5	5	4

### Differential specificity of retigabine and zinc pyrithione between KCNQ channels

**Figure 2:**
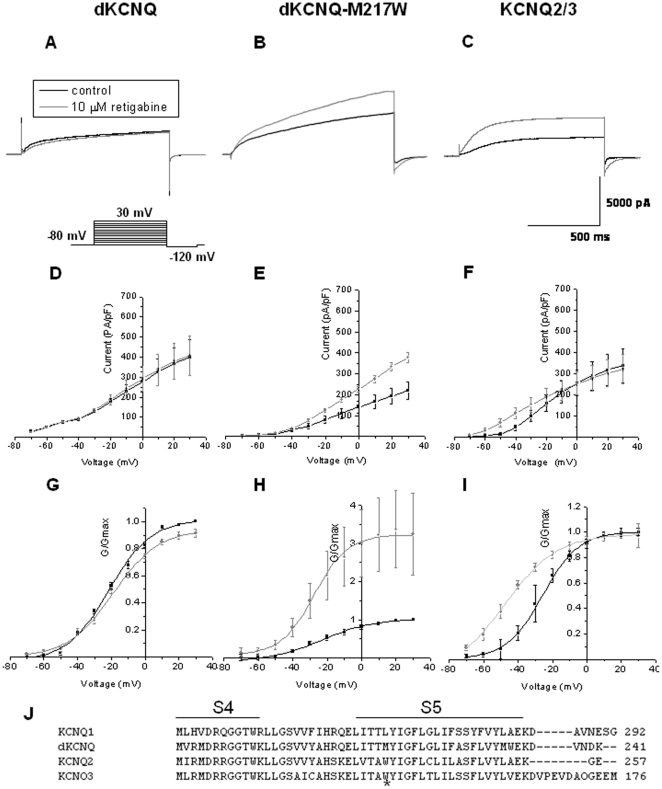
Retigabine displays differential specificity between KCNQ channels with sensitivity conferred by substitution of a single tryptophan residue. (A) Representative traces showing dKCNQ current (black) was insensitive to addition of 10 µM retigabine (grey), no shift in the I-V (D), G-V curve (G) or change (p>0.05) in slope factor (slope factor with retigabine = 18.3±0.8 mV) compared to control (slope factor = 19.7±1.2 mV, n = 5). The same retigabine concentration causes a robust activation of KCNQ2/3 current trace (C) and I-V curve (F) as well as significant leftward shift in the activation curve (I) compared to control. Retigabine did not cause a change (p>0.05) in slope factor for KCNQ2/3 (slope factor = 12.2±1.0 mV) compared to control (slope factor = 10.3±0.7 mV, n = 6). A single amino acid substitution (M217W) in the fifth transmembrane segment of dKCNQ confers sensitivity to retigabine resulting in large current activation (B) at more depolarized voltages on the I-V curve (E) however no leftward shift in the activation curve (H) is seen with the drug, compared to control. There slope factor with drug (slope factor = 21.8±7.0 mV), did not change (p>0.05) compared to control (slope factor = 19.4±7.8 mV, n = 8). The activation was reversible. J) Alignment of KCNQ2, KCNQ3, dKCNQ and KCNQ1 amino acid sequences in the region of the S4 and S5 transmembrane segments. The tryptophan known to be important for retigabine binding [11], [17] is denoted by an asterisk. KCNQ2/3 n = 6, dKCNQ n = 5 and dKCNQ-M217W n = 8.

Retigabine is currently in Phase III clinical trials as an anti-epileptic that specifically activates KCNQ2/3 channels by causing a hyperpolarized shift in voltage activation, reducing neuronal hyperexcitability without affecting KCNQ1/KCNE1 and thus maintaining normal cardiac excitability [17]. A key residue for retigabine binding and activation of KCNQ2 is a tryptophan (W) at position 236 (intracellular side of the fifth transmembrane segment, Figure 2J) [11], [20], [21]. Substitution of this residue (W236L) removes retigabine's shift of the voltage dependence of KCNQ3 channel activation, conversely a L266W substitution at the analogous residue in KCNQ1 (Figure 2J) is sufficient to increase in the maximal apparent P_open_, but does not result in a shift of the activation curve [20]. Addition of 10 µM retigabine (Figure 2C and F) caused a significant (p<0.05) enhancement of the current of approximately 35% at −30 mV as well as a hyperpolarized shift of −24 mV in the activation curve (p<0.05, Figure 2I) of KCNQ2/3 (*V_0.5_* = −47.1±4.5 mV) compared to control (*V_0.5_* = −23.6±2.0 mV, n = 6). dKCNQ was completely insensitive in terms of enhancement of current (p>0.05) at the same concentration of retigabine (Figure 2A), with no shift in the I-V (D) or G-V curve (G) after drug application (*V_0.5_* = −19.8±0.7 mV) compared to control (*V_0.5_* = −21.4±1.0 mV, p>0.05, n = 5) with no significant change in the slope of the activation curve either.

In order to further refine the mode of action and specificity of retigabine on KCNQ subtype biophysics we introduced a W residue at the analogous residue (M217W) of dKCNQ to the retigabine binding site in KCNQ2/3 (Figure 2J). In the absence of drug, the mutation did not change the properties of the channel for instance the *V_0.5_* of the mutant channel (−23.4±5.7 mV) was the same as the wild-type channel (−21.4±1.0 mV) and no change in amplitude of the current was seen for instance at +30 mV the amplitude of the mutant channel (218.8±40.4 pA/pF) was similar (p>0.05) to the wild-type channel (397.2±89.8 pA/pF). Introduction of the tryptophan was sufficient to confer retigabine sensitivity to dKCNQ. 10 µM retigabine caused a robust enhancement of approximately 38% (p<0.0001) of dKCNQ-M217W current at +30 mV (Figure 2B and E), no shift (p>0.05) in the activation curve (*V_0.5_* = −27.5±5.7 mV), compared to control (*V_0.5_* = −23.4±5.7 mV; n = 8) and with no significant change in the slope of the activation curve. Therefore the mechanism of enhancement of retigabine is distinct between the channels, at −30 mV when KCNQ2/3 is enhanced dKCNQ-M217W is not (p>0.05), while at +30 mV when dKCNQ-M217W is enhanced KCNQ2/3 is not (p>0.05). In summary we have shown that a tryptophan at residue 217 is necessary to confer retigabine sensitivity to dKCNQ, suggesting that this residue is especially important amongst the residues that have been implicated mediating the effect of retigabine on KCNQ channels [21].

**Figure 3:**
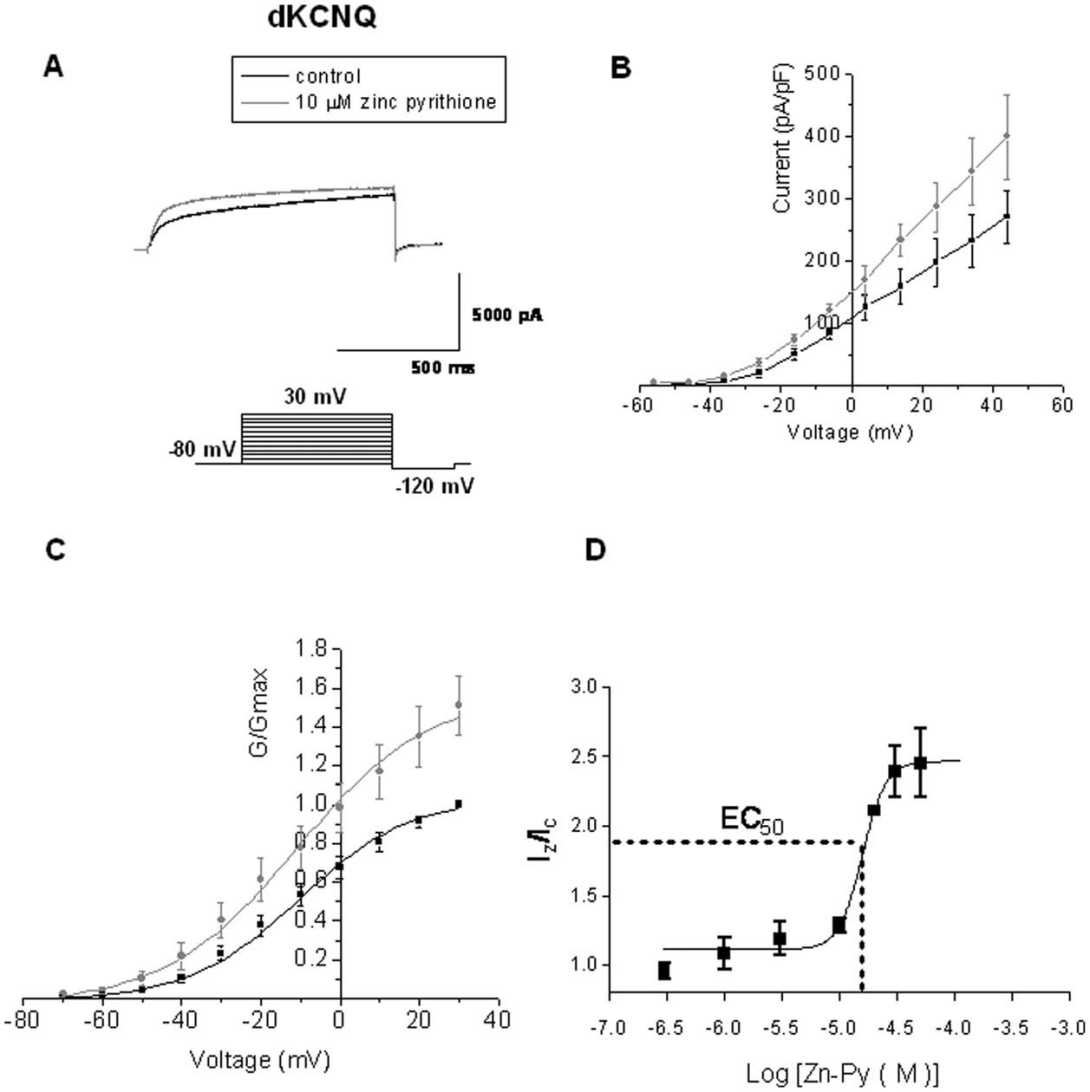
The KCNQ activator, Zinc pyrithione enhances dKCNQ current. Representative traces (A), I-V (B) and G-V (C) plots showing 10 µM zinc pyrithione causes strong activation of dKCNQ compared to control, as has previously been reported for mammalian KCNQ channels [11]. Addition of zinc pyrithione did not change (p>0.05) the slope factor of dKCNQ (slope factor with drug = 18.7±3.9 mV) compared to control (slope factor = 18.1±3.9 mV, n = 5). The activation by zinc pyrithione was reversible. n = 5.

A second KCNQ activator, zinc pyrithione is known to discriminate between channel subtypes, opening KCNQ1, KCNQ2 homomultimers and KCNQ2/3 heteromultimers, but not KCNQ3 homomultimers [11]. Addition of 10 µM zinc pyrithione caused an increase of dKCNQ current of about 20% (at +30 mV, p<0.001) with no shift (p>0.05) in its voltage dependence (*V_0.5_* = −10.9±1.2 mV) compared to control (*V_0.5_* = −10.3±2.4 mV, n = 5) and no significant change in the slope of the activation curve (Figure 3). The EC_50_ of zinc pyrithione on dKCNQ was 15.5±2.4 µM (n≥5) with a Hill coefficient equal to 4.2±0.06 indicating a positive cooperative process with binding of one molecule of zinc pyrithione increasing the binding of subsequent molecules.

### dKCNQ shows conserved inhibition by mammalian KCNQ channel blockers

**Figure 4:**
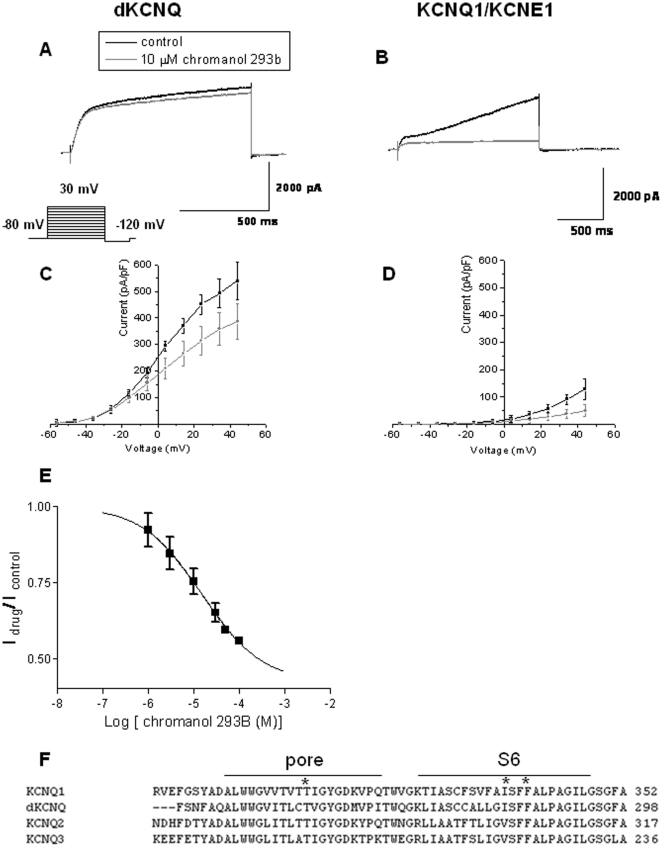
Chromanol 293B show conserved inhibition of *Drosophila* and mammalian KCNQ1/KCNE1 channels. Representative traces showing dKCNQ (A) current (black) was sensitive to block by 10 µM chromanol 293B (grey), this is also reflected in the downward shift of the I-V curve (C). Traces (B) and I-V curves (D) showing KCNQ1/KCNE1 was sensitive to block by 10 µM chromanol 293B. (E) Dose response curve for chromanol 293B effect on dKCNQ current. The block was reversible. F) Alignment of KCNQ1, dKCNQ, KCNQ2 and KCNQ3 channels protein sequence in the region of the pore loop and S6 transmembrane segment. The important residues for the chromanol 293B binding site are marked by asterisks [4]. dKCNQ n = 8 and KCNQ1/KCNE1 n = 4.

Chromanol 293B inhibits the cardiac I_Ks_ potassium current and is specific for KCNQ1/KCNE1 as opposed to KCNQ2 [4]. Three critical residues (T312, I337 and F340) forming the putative drug-binding site in the pore and sixth transmembrane domain [4] are conserved between KCNQ1 and dKCNQ (Figure 4F). Chromanol 293B was found to be effective at blocking dKCNQ at 30 mV causing an approximately 30% reduction in current (p<0.05, Figure 4A and C, n = 8) and blocking KCNQ1/KCNE1 at 30 mV causing an approximately 56% decrease in current (p<0.05, Figure 4B and D, n = 4) without causing a change in the activation curve of the respective channels (Figure S1A–B). The IC_50_ for dKCNQ was 13.0±3.6 µM with a Hill coefficient of 0.7±0.1 (n≥8, Figure 4), similar to IC_50_ for KCNQ1/KCNE1 of about 6.9±0.5 µM [4] and suggesting chromanol 293B acts by a non-cooperative process whereby each molecule bound decreases the affinity of binding of a subsequent molecule of the drug.

**Figure 5:**
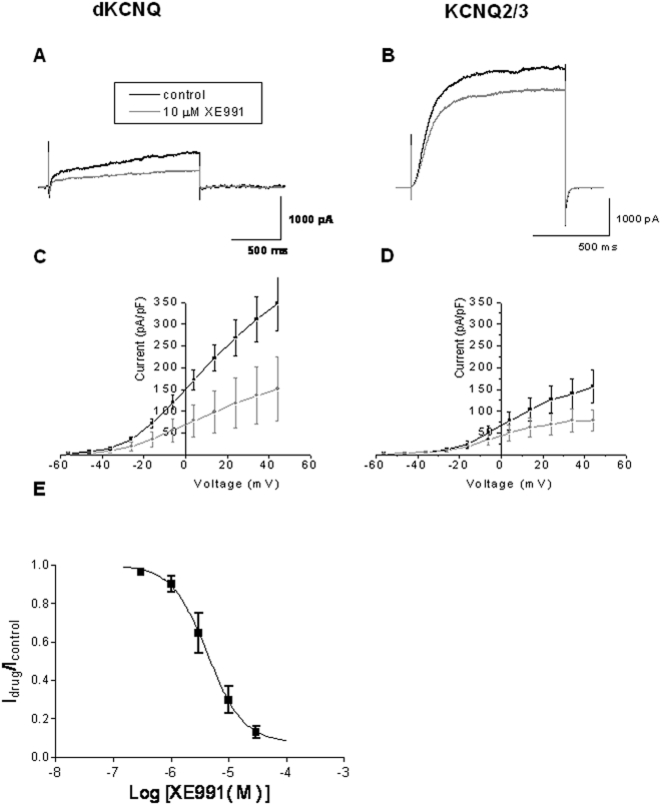
XE991 show conserved inhibition of *Drosophila* and mammalian KCNQ2/3 channels. Representative traces showing dKCNQ (A) current (black) was sensitive to block by 10 µM XE991 (grey), this is also reflected in the downward shift of the I-V curve (C). Traces (B) and I-V curves (D) showing KCNQ2/3 was sensitive to block by 10 µM XE991. The block was reversible. (E) Dose response curve for XE991 effect on dKCNQ current. dKCNQ n = 6 and KCNQ2/KCNQ3 n = 6.

The M-current is known to be blocked by XE991 and linopirdine in both native neurons and when heterologously expressed in cells [7], [18]. Addition of 10 µM of XE991 similarly blocked dKCNQ (p<0.05, Figure 5A and C, n = 6) and KCNQ2/3 currents (p<0.05, Figure 5B and D, n = 6) reducing their currents by about 50% at 30 mV, with little change in activation curves of the channels (Figure S1C–D). The IC_50_ of XE991 for dKCNQ was 5.5±1.1 µM with a Hill coefficient of 1.4±0.5 (n≥8) which is slightly higher value compared to 0.6±0.1 µM for KCNQ2/3 and 0.75±0.075 µM for KCNQ1 reported previously [7]. A Hill coefficient equal to approximately one suggests a non cooperative process, whereby the affinity of the molecule for a channel is not dependent on whether or not other molecules are already bound. Likewise the XE991 analogue, linopirdine, also showed inhibition of the mammalian M-current by about 40% and dKCNQ by 30% (p<0.05, Figure S2).

## Discussion

In order to study the function of KCNQ channels further and develop a new genetic model for KCNQ channelopathies we have characterised for the first time the electrophysiological and pharmacological properties of dKCNQ in comparison to the mammalian cardiac and neuronal KCNQ channels in order to validate such an approach. Whereas the human genome contains one cardiac (KCNQ1) and four neuronal (KCNQ2-5) channels in this subfamily [2], *Drosophila* has a single channel (dKCNQ) broadly expressed in both the cardiac and nervous system of the fly fulfilling all the functions of the duplicated mammalian KCNQ genes, with evolutionarily conserved electrophysiological and pharmacological features of both the cardiac and neuronal KCNQ channels. Underlying this functional conservation is a high degree of molecular conservation between the human KCNQ channels and the ancestral KCNQ in *Drosophila*, with the neuronal KCNQ channels being more related to the dKCNQ than the cardiac channel. The diversity of KCNQ channels in mammals has made studying these channels in native tissue problematic making characterisation of functional consequence of removal of a given KCNQ channel at the whole animal level difficult due to genetic redundancy or compensation by the remaining KCNQ channels. A possible solution to this problem is to use the power of *Drosophila* genetics, flies with a complete lack of any KCNQ channels are adult viable and have been shown to have key hallmarks of KCNQ channelopathies [16]. We found that dKCNQ encodes a slowly activating and slowly deactivating voltage gated K^+^ current with activation in the sub-threshold voltage range (from about −60 mV) similar to the M-current, this being a key feature allowing these channels to be important regulators of neuronal excitability and why loss of function mutations cause a form of epilepsy and are associated with neuropathic pain [2], [13], [17], [18]. Again this suggests that *Drosophila* would be a valid model to understand these neural KCNQ channelopathies and screen for new modulators. We found that the electrophysiological properties of dKCNQ are more similar to the M-current encoded by mammalian KCNQ2/3 but intermediate in kinetics, current amplitude and voltage activation between the neuronal and cardiac channel. The values we report for dKCNQ are comparable to those of a previous study [15], with our V_0.5_ being slightly more negative and our deactivation values being a little faster. These small differences may result from the different expression systems (HEK this study as compared to CHO cells in the previous study) and slightly different solutions used in each study. The V_0.5_ (−15.3±1.4 mV) for KCNQ2/3 is very similar to the value previously reported (−17.3±2.2 mV; [17]). The V_0.5_ of 19.4±1.2 mV for KCNQ1/KCNE1 is however more positive compared to −12.5±1.1 obtained previously [17], this could be due to the fact that the voltage step duration we used may not have been sufficient for the current to reach the maximal activation. Overall the dKCNQ properties we have described are similar to those reported in the study of Wen and colleagues who showed that dKCNQ like KCNQ2/3 could be suppressed by muscarinic acetylcholine receptor agonist and hence displays the hallmark of an M-current [15].

In terms of pharmacology, we show dKCNQ display conserved sensitivity to the mammalian cardiac and neuronal KCNQ drugs with the classic M-current blockers such as linopirdine and XE991 being effective in inhibiting dKCNQ. Conversely specific enhancers of neuronal KCNQ channels such as retigabine are effective as anticonvulsants by enhancing KCNQ2-5 currents while not disrupting the cardiac KCNQ1 currents [11], [17]. We found dKCNQ was insensitive to retigabine, like KCNQ1 which also lacked the tryptophan necessary for retigabine binding. We therefore introduced this residue into dKCNQ (M217W) and found this was sufficient to allow retigabine to increase the dKCNQ current. Substitution of this single residue in dKCNQ was not however sufficient to confer a hyperpolarizing shift in voltage activation in the presence of retigabine. The magnitude of the hyperpolarizing shift caused by retigabine was greatest for KCNQ3, followed by KCNQ2/3, KCNQ2, KCNQ4 and KCNQ1-L266W, each resulting in an increase in P_open_ probability [17], [18]. The non-significant shift (−3 mV) of the dKCNQ activation curve is most similar to the shift (−3 mV) seen for homomeric KCNQ4 [17]. Our data suggest that there are additional residues required to allow the retigabine induced hyperpolarizing shift in activation that are not present in dKCNQ but are present in mammalian neuronal KCNQs. Recently the retigabine binding site has been redefined highlighting the importance of additional residues for hyperpolarizing shift in activation of KCNQ channels [21], these could be candidate residues to confer this sensitivity to dKCNQ.

Activation of K^+^ channels by chemical openers is generally rare and therefore mechanistically revealing. Furthermore, KCNQ openers have the potential to treat epilepsy, cardiac arrhythmias, neuropathic pain, anxiety and neurodegenerative diseases [2], [11], [13], [18]. Zinc pyrithione is another neuronal KCNQ opener that binds at an independent site to retigabine (therefore, for each KCNQ tetrameric channel there are four zinc pyrithione and four retigabine binding sites) with coapplication of the drugs allowing rescue of KCNQ2 benign familial neonatal convulsions causing mutations to wildtype [11]. We therefore tested if dKCNQ was sensitive to activation by zinc pyrithione and found the drug gave an enhancement of current with a Hill coefficient that suggests that the first molecule bound to the channel promotes the binding of subsequent molecules to the channel complex, this is similar to values for KCNQ2 suggesting that their binding sites may show some conservation [11]. Further work will be required to determine these conserved residues, however we have demonstrated that dKCNQ can be used to study subtype specificity of KCNQ chemical modulators and that *Drosophila* has the potential to be used to screen and characterise the function of new neuronal KCNQ enhancers and blockers.

KCNQ has also been shown to have conserved cardiac functions between *Drosophila* and human [16], we therefore wished to see if potential class III antiarrhythmics that inhibit cardiac I_Ks_ potassium channels were also effective on dKCNQ currents. We found that chromanol 293B [4] similarly blocked both the mammalian cardiac and *Drosophila* KCNQ with the three critical residues (KCNQ1 T312, I337 and F340) forming the putative drug-binding site in the pore and sixth transmembrane domain being conserved in both channels. Previous work has shown that chromanol 293B specifically acts on the native slow delayed current (I_Ks_) mediated by KCNQ1/KCNE1 subunits but has little effect on homomeric KCNQ1 or on KCNQ2-5 (4). The homomeric KCNQ1 channel mediates a rapidly activating and slowly deactivating K^+^ current which is not a native current normally found in cardiac tissue [19].

The mechanisms underlying development and function of the heart are basically conserved between *Drosophila* and human with KCNQ channels having a central and conserved role in the hearts of both types of animal [16]. Because *Drosophila* has a separate means to distribute oxygen via its tracheal system, genetic or drug manipulations that cause extreme cardiac pathology can be characterised which would be immediately lethal in mammalian systems [22]. We have gone on to show that dKCNQ electrophysiology and pharmacology also show conservation with human KCNQ1/KCNE1 channels. This allows *Drosophila* to be used as a valid model to screen and understand mechanism of actions of new KCNQ based therapies for cardiac channelopathies. One important advantage of such a model is it will be possible to high-throughput screen for either new chemical or genetic modulators of KCNQ pathological states at the whole organism level allowing the signalling networks underlying cardiac arrhythmias or epilepsy [22]–[24], to be interrogated by *Drosophila* molecular genetics. In summary the ancestral KCNQ channel in *Drosophila* displays conserved electrophysiology and accompanying pharmacology intermediate between the cardiac and neuronal KCNQ allowing it to serve all the functions of the duplicated mammalian KCNQ channels expressed in the cardiac and nervous systems.

## Materials and Methods

### Sequence comparison

Genomic database searches were performed with *Drosophila* melanogaster RE26469 full-length *KCNQ* cDNA (FlyBase ID FBcl0234919) using the WU-BLAST (basic local alignment search tool) server at EMBL-EBI (European Bioinformatics Institute).

### DNA reagents

*Drosophila KCNQ RE26469* cDNA (Flybase FBgn0033494, vector: *pIRES2-EGFP*; [15]), rat *KCNQ2* cDNA (GenBank accession no: AAC36722; vector: *pcDNA3.1*; [25]), rat *KCNQ3* cDNA (GenBank accession number AC79846; vector: *pcDNA3.1*; [25]) and human *KCNQ1* cDNA co-expressed with *KCNE1* cDNA (accession no: AF000571; vector: *pIRES1-CD8*; [3]) were used for all electrophysiological and pharmacological studies. The *Drosophila KCNQ* point mutant was made by site-directed mutagenesis (QuickChange, Stratagene) and was verified by sequencing.

### Cell culture

cDNA were expressed in HEK (ECACC, Salisbury, UK) cells for electrophysiological studies [25]. Cells were dissociated for propagation with PBS-based solution containing EDTA and kept at 37°C on Dulbecco's modified eagle medium supplemented with 10% fetal calf serum and 0.1% penicillin/streptomycin (Invitrogen). The cells were plated onto 35 mm dishes 48 hours before transfection for electrophysiological studies. Transfection of cells was made with polyethylenimine (PEI, Johnson Matthey) by adding cDNA channel plasmids (1 µg dKCNQ, 1 µg KCNQ2 and KCNQ3 in a ratio 1∶1, 1 µg KCNQ1 and 2 µg KCNE1 in a ratio 1∶2) and coexpressing a plasmid containing *green fluorescent protein* (*GFP*) cDNA in ratio 1∶1. GFP positive cells were selected for subsequent electrophysiological recordings 12–24 hours of transfection.

### Electrophysiology and pharmacology

Whole-cell voltage-clamp recordings were made from single, uncoupled cells at room temperature using an Axopatch 200A amplifier (Axon Instruments). Data was acquired using PulseFit and Pulse software (HEKA Elektonik). Fire-polished electrodes (3–4 MΩ) were pulled from borosilicate glass and containing the following for the retigabine studies (in mM): 140 KCl, 0.5 MgCl_2_, 2 CaCl_2_, 5 EGTA, and 10 HEPES, at pH 7.2 with KOH. This gave an internal solution containing 100 nM free Ca^2+^. The external bathing solution was constantly perfused and contained (in mM): 145 NaCl, 5 KCl, 2 MgCl_2_, 2 CaCl_2_, 10 HEPES at pH 7.2 with KOH, and 310–320 mOsmol. The previous solutions were adapted from the work on dKCNQ channels in CHO cells [15]. During the course of the study, we started to use solutions especially adapted for recording KCNQ currents expressed in HEK cells [25] this improved the quality of our recordings. However, the difference in amount of ions present in the two sets of solutions gave a ∼10 mV different in V_0.5_ value between recordings. For all other experiments the following internal solution was used (in mM): 130 K aspartate, 0.1 EGTA, 5 HEPES (Na), 3 NaOH, 1.5 MgCl_2_, 20 KCl, 6.19 CaCl_2_ (calculated free [Ca^2+^] = 80 nM), 1.5 NaATP at pH 7.2 with NaCl, the osmolarity was approximately 305 mOsm. The external solution bath was composed of (mM): 144 NaCl, 2.5 KCl, 1.2 MgCl_2_, 2.5 CaCl_2_, 10 HEPES (acid), 10 D-glucose at pH 7.2 with NaCl, the osmolarity was approximately 315 mOsmol. The liquid junction potential was calculated as −14 mV using the application JPCcalc of pClamp software (Axon instruments) and was subtracted from voltages to give the x-axis values plotted. The currents were measured after capacitance and series resistance compensation (>95%), filtered at 1 kHz using an 8-pole Bessel filter (Frequency Devices), and sampled at 5 kHz using Pulse (HEKA). Recordings were subjected to a P/4 leak subtraction protocol [26], which consisted of four preceding hyperpolarizing prepulses to a voltage one quarter the size of the test pulse that was to follow starting at −80 mV where the currents of interest are inactive. Drugs used in the study, retigabine, linopridine, XE991 (10,10-*bis*(4-Pyridinylmethyl)-9(10*H*)-anthracenone), chromanol 293B and zinc pyrithione, were obtained either from Sigma or Tocris. Stock solutions of drugs were made in water, alcohol, or dimethyl sulfoxide. The concentration of solvents added to the cells had no effect by itself. Extracellular application of the drugs was performed in the recordings presented.

To show the effect of drug application at maximum depolarization, traces were generated using a single pulse protocol where cells were held at −80 mV for 100 ms, stepped to +30 mV then after 1 s, stepped down to −120 mV for 250 ms. To generate I-V and G-V curves the following multi-step protocol was used, cells were held at −80 mV for 100 ms, then stepped in increments of 10 mV from −80 mV to +30 mV then after 1.3 s, before stepping down to −120 mV for 250 ms. KCNQ1/KCNE1 expressing cells which were depolarized from −80 mV to 30 mV then after 2.5 sec were stepped down to −120 mV for 250 ms. In order to construct the current-voltage (I-V) plots, currents were measured at the end of the sweep at the maximum of the current for each step. I-V curves were plotted as normalised current in pA/pF against membrane potential in mV. The cell capacitance was obtained by nullifying the capacitive transients in the test pulse. All mean current values are presented as ± standard error of the mean (SEM).

Activation curves were calculated from the tail current at −120 mV at the end of each test pulse. Normalised tail currents were plotted against voltage to create the activation curve. For the retigabine and zinc pyrithione enhancers the current amplitude was measured at each voltage step and normalised to the maximum tail current amplitude at 30 mV in absence of the drugs. Activation curves were fit to the Boltzmann equation G/Gmax = 1/{1+exp[(V_0.5_−Vm)/S]}, where Vm is the membrane voltage, V_0.5_ is the voltage at half-maximal activation, and S is a slope factor at Vm = V_0.5_ [27]. Mean V_0.5_ values are shown ± S.E.M. The exponential time course of activation and deactivation curves were fitted using PulseFit software (HEKA Elektonik). The activation curve was fitted with double exponentials for all three channels from the start of the current at the beginning of the pulse to the point of peak current activation. A double exponential provided the best fit for the dKCNQ deactivation curve, while a single exponential gave the best fit of the KCNQ2/3 and KCNQ1 channels. The exponential was fitted after the peak of the current and at a point at the end of the sweep.

For concentration response relationships data points representing fractional current remaining (*I/I_cont_*) were fit with a variable Hill coefficient equation of the form: *I*/*I_control_ = A_1_+*(A_2_−A_1_)/1+10^(logEC50−[X])nh^, where I is the recorded macroscopic current, I_cont_ the current in control conditions, A_1_ the minimum fraction of current remaining, A_2_ the maximum fraction of current remaining, EC_50_ the antagonist/agonist concentration that provokes a response halfway between baseline and maximum, X is the logarithm of agonist/antagonist concentration and n_h_ the Hill coefficient. Unless otherwise stated the fitting regime was performed on data from individual experiments and the fit parameters quoted are of the mean ± SEM of the individual fits. The fractional block and the fractional enhance were calculated with the following equation: 1−(*I_blocked_/I_control_*) and (*I_enhanced_/I_control_*)−1. The curves displayed in the figures are representative curve fits to the mean data. Data were analysed using Graphpad Prism with Student's paired *t* test. Currents size and its regulation by drugs were measured from the amplitude of the activation current recorded at +30 mV. Statistical analysis was performed making a comparison between drug and control amplitude with a paired *t* test.

## Supporting Information

Figure S1 **The KCNQ blockers: chromanol 293B and XE991 do not cause a change in voltage activation of the** ***Drosophila*** **and mammalian KCNQ channels.** The V_0.5_ and slope factor of activation curves of dKCNQ with chromanol 293B application (V_0.5_ = −7.4±3.6 mV; slope factor = 16.3±3.2 mV) were similar (p>0.05) to control (V_0.5_ = −5.3±3.4 mV; slope factor = 15.5±2.2 mV, n = 8). Likewise G-V curves (B) for KCNQ1/KCNE1 with 10 µM chromanol 293B application (V_0.5_ = 22.1±6.8 mV; slope factor = 14.7±1.9 mV) overlapped with control (V_0.5_ = 25.2±2.5 mV; slope factor = 13.3±0.6 mV, n = 4), with no changes in V_0.5_ or slope factor (p>0.05). The activation curve of dKCNQ (C) with XE991 (V_0.5_ = −6.8±1.8 mV and slope factor = 14.7±2.1 mV) was similar to control (V_0.5_ = −6.0±0.8 mV; slope factor = 12.2±0.5 mV, n = 6), with no changes in V_0.5_ or slope factor (p>0.05). The KCNQ2/3 activation curve (D) with XE991 gave values for V_0.5_ = −22.0±1.8 mV and slope factor = 11.1±1.1 mV neither of which were significantly (p>0.05) shifted compared to control (V_0.5_ = −13.2±0.5 mV; slope factor = 14.3±1.8 mV, n = 6).
(TIF)

Figure S2 **The KCNQ blocker: linopirdine shows conserved inhibition of** ***Drosophila*** **and mammalian KCNQ channels.** Representative traces (A) and I-V curve (C) showing dKCNQ current (black) is sensitive to block (at 30 mV, p<0.01) by 10 µM linopirdine (grey), similar inhibition (p<0.05) of KCNQ2/3 is seen (B and D). Activation curves of dKCNQ (C) with linopirdine (V_0.5_ = −5.7±3.7 mV and slope factor = 18.5±3.0 mV) were not different from control (V_0.5_ = −10.0±2.0 mV; slope factor = 14.5±1.5 mV, n = 5) in terms of V_0.5_ or slope factor (p>0.05). The KCNQ2/3 activation curve (D) with linopirdine gave values for V_0.5_ = −18.5±1.0 mV and slope factor = 8.7±0.9 mV neither of which were (p>0.05) shifted compared to control (V_0.5_ = −14.0±1.4 mV; slope factor = 12.8±1.3 mV, n = 5). The block was reversible.
(TIF)
