# Supplementary figures and images for: Drosophila KCNQ Channel Displays Evolutionarily Conserved Electrophysiology and Pharmacology with Mammalian KCNQ Channels
Source: PLoS One. 2011 Sep 7;6(9):e23898. doi: 10.1371/journal.pone.0023898 (PMC3168433; doi:10.1371/journal.pone.0023898)

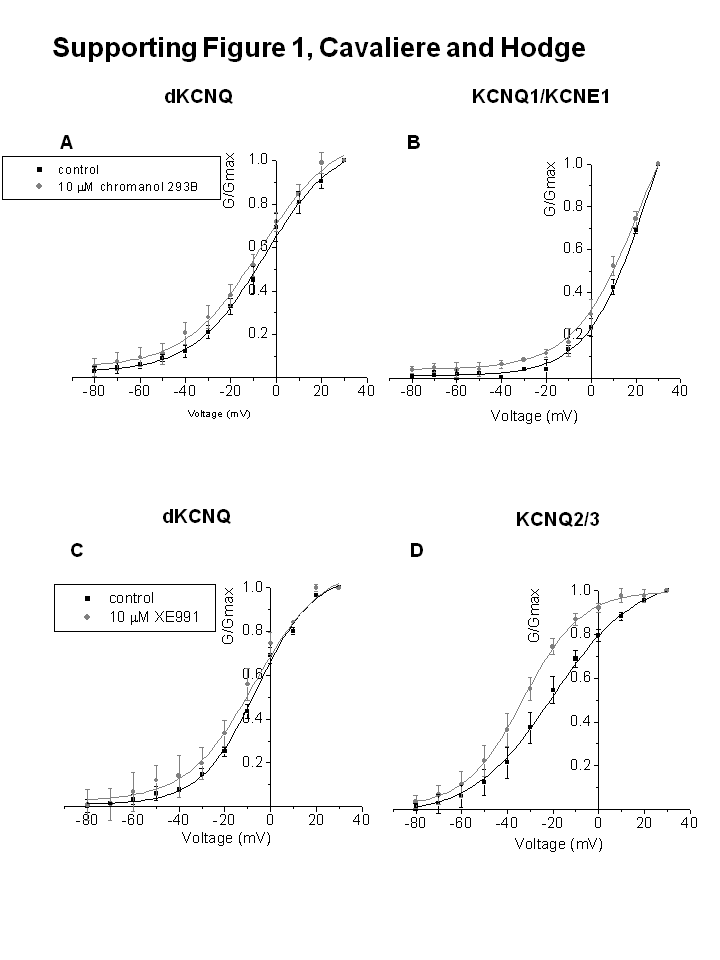

Supplement: Figure S1 — The KCNQ blockers: chromanol 293B and XE991 do not cause a change in voltage activation of the Drosophila and mammalian KCNQ channels. The V0.5 and slope factor of activation curves of dKCNQ with chromanol 293B application (V0.5 = −7.4±3.6 mV; slope factor = 16.3±3.2 mV) were similar (p>0.05) to control (V0.5 = −5.3±3.4 mV; slope factor = 15.5±2.2 mV, n = 8). Likewise G-V curves (B) for KCNQ1/KCNE1 with 10 µM chromanol 293B application (V0.5 = 22.1±6.8 mV; slope factor = 14.7±1.9 mV) overlapped with control (V0.5 = 25.2±2.5 mV; slope factor = 13.3±0.6 mV, n = 4), with no changes in V0.5 or slope factor (p>0.05). The activation curve of dKCNQ (C) with XE991 (V0.5 = −6.8±1.8 mV and slope factor = 14.7±2.1 mV) was similar to control (V0.5 = −6.0±0.8 mV; slope factor = 12.2±0.5 mV, n = 6), with no changes in V0.5 or slope factor (p>0.05). The KCNQ2/3 activation curve (D) with XE991 gave values for V0.5 = −22.0±1.8 mV and slope factor = 11.1±1.1 mV neither of which were significantly (p>0.05) shifted compared to control (V0.5 = −13.2±0.5 mV; slope factor = 14.3±1.8 mV, n = 6). (TIF) [file pone.0023898.s001.tif]

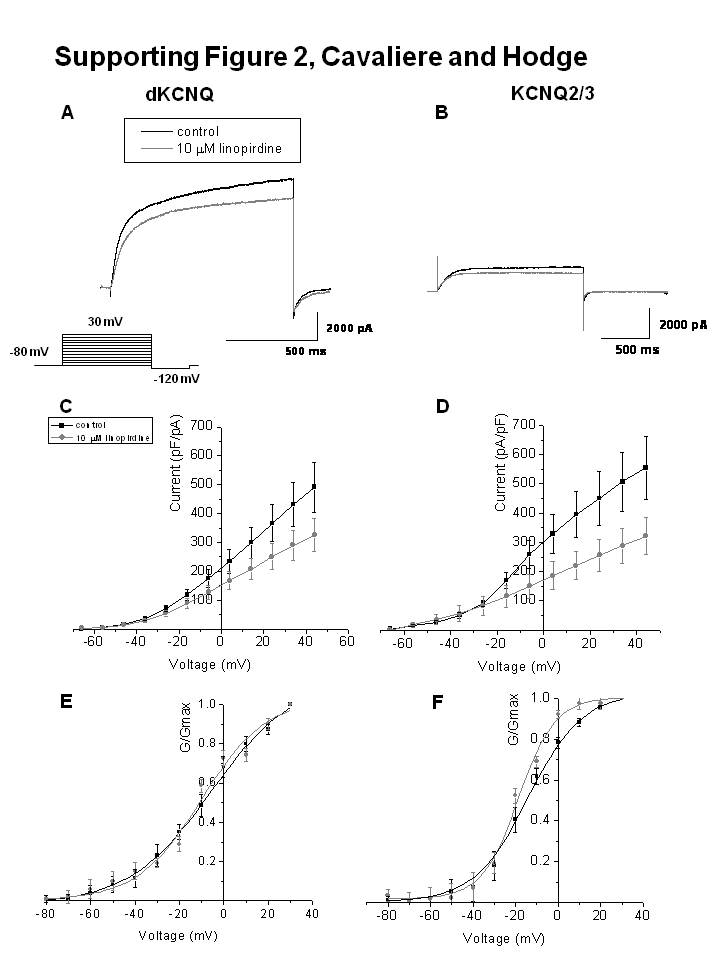

Supplement: Figure S2 — The KCNQ blocker: linopirdine shows conserved inhibition of Drosophila and mammalian KCNQ channels. Representative traces (A) and I-V curve (C) showing dKCNQ current (black) is sensitive to block (at 30 mV, p<0.01) by 10 µM linopirdine (grey), similar inhibition (p<0.05) of KCNQ2/3 is seen (B and D). Activation curves of dKCNQ (C) with linopirdine (V0.5 = −5.7±3.7 mV and slope factor = 18.5±3.0 mV) were not different from control (V0.5 = −10.0±2.0 mV; slope factor = 14.5±1.5 mV, n = 5) in terms of V0.5 or slope factor (p>0.05). The KCNQ2/3 activation curve (D) with linopirdine gave values for V0.5 = −18.5±1.0 mV and slope factor = 8.7±0.9 mV neither of which were (p>0.05) shifted compared to control (V0.5 = −14.0±1.4 mV; slope factor = 12.8±1.3 mV, n = 5). The block was reversible. (TIF) [file pone.0023898.s002.tif]
